# Utilization of sugarcane bagasse waste by *Aspergillus welwitchiae* SVUAw9 for production, purification, and characterization of cold-active xylanase under solid-state fermentation conditions

**DOI:** 10.1186/s12934-026-03025-7

**Published:** 2026-05-19

**Authors:** Asmaa S. Yassein, Walaa S. Ahmed, Ahmed H. Elsayed, Mohamed A. Hussein, Osama A. M. Al-Bedak

**Affiliations:** 1 Department of Botany and Microbiology, Faculty of Science, Qena University, Qena, 83523 Egypt; 2 Assiut University Mycological Centre, Assiut University, Assiut, 71511 Egypt; 3 ERU Science & Innovation Center of Excellence, Egyptian Russian University, Badr city, Cairo, 11829 Egypt

**Keywords:** Cold-adapted, *Bagasse*, Enzymes, Fungi, Lignocellulose, Plant-biomass-derived waste, Xylanase

## Abstract

*Aspergillus welwitschiae* strain SVUAw9 was isolated from sugarcane bagasse and identified by the sequencing of the calmodulin (CaM) gene, was found to exhibit substantial cold-active xylanase activity in this study. Under solid-state fermentation conditions, the strain can utilize a range of agro-industrial residues as substrates to produce cold-active xylanase. After 14 days at pH 7 and 10 °C using ammonium chloride as the nitrogen source, sugarcane bagasse was the most productive substrate, generating 106.93 ± 12.1 U/gram dry substrate (gds), followed by bean straw, corn cob, and date palm leaves which produced 57.82 ± 8.27, 57.75 ± 7.44, and 38.87 ± 6.15 U/gds, respectively. Conversely, the substrate exhibiting the lowest production was rice husk, producing 29.32 ± 4.88 U/gds. Using Trilite MC 08 and Sephacryl S-200 columns, the xylanase was purified 75.87 times, yielding a single band at approximately 71 kDa. At pH 4.0 and 30 °C, the maximum activity of 156.46 ± 12 U/mg was achieved. Co(NO₃)₂, MnSO₄, and NiSO₄ markedly enhanced enzyme activity, resulting in residual activity increases of 152.94 ± 11.54%, 152.94 ± 9.58%, and 134.12 ± ठर 7.66%, respectively, with specific activities of 239.3 ± 18, 239.3 ± 15, and 209.84 ± 12 U/mg. Km and Vmax for the pure xylanase were determined as 0.1 ± 0.005 mg/mL and 144.9 ± 7.14 µmol/min, respectively. The purified xylanase could degrade oat spelt xylan, corn cob xylan, xylan, Birchwood xylan, maize stalk xylan, carboxymethyl cellulose (CMC), beechwood and microcrystalline cellulose (MCC), resulting in activities of 156 ± 12, 108 ± 7, 82 ± 6, 79 ± 5, 62 ± 4, 22 ± 1.5, and 38 ± 3 U/mg, respectively.

## Introduction

Global agricultural activities generate more than 5 billion metric tons of lignocellulosic biomass [1, 2]. Lignocellulosic biomasses include barley bran, barley hay, bean straw, date palm leaves, rice bran, rice straw, sugarcane bagasse, wheat bran, wheat straw, vegetable and fruit remains, and cotton leaf scraps. These wastes have indisputably resulted in environmental disturbances, likely due to the challenges associated with their correct disposal [2, 3]. Consequently, it is imperative to mitigate the detrimental impacts of these wastes on the environment while concurrently repurposing them into feasible industrial and commercial products [4]. Sugarcane bagasse, a fibrous residue, consists of approximately 32–45% cellulose, 20–32% hemicellulose, 17–32% lignin, 1.0–9.0% ash, and other compounds [5, 6]. Moreover, it is regarded as a viable biomass substitute to produce energy, carbohydrates, enzymes, and second-generation biofuels [4, 7]. Microorganisms are often utilized as a source of enzymes due to their rapid and extensive culture capabilities. Microbial proteins typically exhibit greater stability than enzymes derived from alternative sources, such as plants or animals [8].

Xylan, mannan, galactan, and arabinan are examples of hemicelluloses, which are essential polymeric components of lignocellulosic biomass. Xylose is the principal monomer in most hemicelluloses, constituting the main component of xylan. Xylan, the second most prevalent polysaccharide in nature, consists of xylose units linked by 1,4-β-glycosidic linkages, forming strands along the plant backbone [9, 10]. Xylan is in the secondary cell wall at the juncture of lignin and cellulose, maintained by covalent and non-covalent bonds, to uphold the structural integrity of the cell wall and the cohesion of the fibers [11, 12]. A variety of xylanolytic enzymes, including α-D-glucuronidase, endo-β−1,4-D-xylanase, and α-l-arabinofuranosidase, are essential for the hydrolysis of xylan [13, 14]. Endo-β−1,4-xylanases primarily cleave the internal β−1,4-xylosidic linkages of the xylan backbone, generating xylooligosaccharides [15–17]. These oligomers are further degraded by β-xylosidases, which remove terminal xylose units and convert xylooligosaccharides into monomeric xylose.

The endo-β−1,4-D-xylanase (EC 3.2.1.8) cleaves the β−1,4-glycosidic linkages within the xylose chain [14]. Xylanases are classified into glycosyl hydrolase (GH) families 5, 7, 8, 10, 11, 30, and 43 based on their catalytic domain sequences [13, 18, 19]. Wong et al. [20] categorized xylanases into two principal groups based on their physicochemical characteristics: low–molecular-weight enzymes (< 30 kDa) with basic isoelectric points, and high–molecular-weight enzymes (> 30 kDa) with acidic isoelectric points [13, 21, 22]. Large-molecular-weight xylanases are distributed across several glycoside hydrolase (GH) families beyond the classical GH10 group, which was originally associated with high molecular mass. Numerous GH5 xylanases exceed 30 kDa, including the *Aeromonas punctata* (caviae) ME-1 enzyme XynD, reported at approximately 58 kDa [23], as well as multi-domain GH5 enzymes from *Erwinia chrysanthemi* and *Trichoderma reesei*, which surpass 30 kDa due to the presence of additional structural domains [24].

GH8 xylanases are also consistently high in molecular mass, with both the cold-adapted xylanase from *Pseudoalteromonas haloplanktis* TAH3a and the *Bacillus* sp. KK-1 xylanase measured at 45–46 kDa [25–29]. GH10 xylanases, long recognized as high-molecular-weight enzymes, typically possess large catalytic domains and acidic pI values, and many characterized representatives fall well above the 30 kDa threshold [13, 30–33]. Additional high-MW xylanolytic enzymes include GH7 endoglucanase I (Cel7B/EGI) from *Trichoderma reesei*, which exhibits xylanase activity despite being a non-specific endo-β−1,4-glucanase and has a high molecular weight along with a low isoelectric point [34–36], as well as the GH43 enzyme XYND from *Paenibacillus polymyxa*, which has a molecular weight of approximately 64 kDa and displays both xylanase and α-L-arabinofuranosidase activities [37]. These examples collectively demonstrate that high-molecular-weight xylanases occur across multiple GH families—including GH5, GH8, GH10, GH7, and GH43—and highlight why nearly 30% of currently known xylanases, particularly those of fungal or multi-domain origin, fall outside the two-group classification model proposed by Wong et al. [20].

A diverse microbial community, encompassing filamentous fungi, bacteria, and yeasts, has been recognized for its capacity to produce xylanase [20, 38–40]. From an industrial perspective, fungi are notable for their enhanced extracellular xylanase production and the secretion of many auxiliary enzymes essential for debranching substituted xylans [41]. Xylanases provide significant biotechnological potential to produce liquid fuels and chemicals from lignocellulose, in addition to facilitating environmentally sustainable technologies in the paper and pulp, feed, and food sectors [42, 43]. Recently, there has been a significant increase in research concerning the biotechnological uses of cold-active enzymes [44]. Conversely, cold-active xylanases exhibit thermostability at elevated temperatures and demonstrate optimal activity at low to moderate temperatures. Cold-active xylanases can preserve energy by limiting fermentation and microbial proliferation, hence preserving the quality of components and products in industrial applications [13, 19, 45, 46]. The limited understanding of cold-active xylanases stems from the fact that they remain significantly under-investigated, and therefore their true prevalence in nature is still largely unknown. In contrast to their mesophilic (40–60 °C) and thermophilic (> 60 °C) counterparts, cold-active enzymes exhibit optimal functionality between 0 and 40 °C, maintaining a consistent reaction rate at these lower temperatures [47, 48].

Solid-state fermentation (SSF) technique is advocated as it promotes elevated enzyme concentrations while requiring minimal energy expenditure [49]. Effective microorganisms must be selected for xylanase production, followed by the optimization of medium’ components and growth conditions to maximize xylanase production, as substantial amounts of xylanase enzymes are required for industrial applications. Accordingly, *Aspergillus welwitschiae* SVUAw9 was used in this study to optimize xylanase production in submerged fermentation (SmF). SSF was utilized to generate economical xylanase by *A. welwitschiae* SVUAw9, employing sugarcane bagasse as the substrate. Subsequently, the xylanase enzyme was purified and characterized.

## Materials and methods

### Isolation of*Aspergillus* strain

The *Aspergillus* sp. isolate SVUAw9 in this investigation was isolated from a sugarcane bagasse sample collected from local market in Qena Governorate, Egypt. The direct plate technique was employed, in which five segments of the sugarcane bagasse sample were positioned on the surface of Petri dishes with Czapek’s agar (CzA) enriched with 50 mg/L Rose Bengal [50]. The plates were subsequently incubated at 25 °C for a duration of seven days. The cultivated fungus was subsequently isolated, purified, and maintained as pure cultures at −86 °C in a 20% glycerol solution and on cotton balls [51].

### Morphological and molecular identification of the*Aspergillus* sp. SVUAw9

The isolate of *Aspergillus* sp. SVUAw9 used in this study was morphologically identified based on its macroscopic and microscopic characteristics using some relevant [52]. In a three points pattern spore suspension obtained from a seven-day-old culture was used to inoculate 9-cm Petri dishes containing CzA, malt extract agar (MEA), and Czapek’s yeast extract agar (CYA) [53]. Plates were then incubated in the dark at 25 °C for 7 days. The microscopic characteristics were examined from the MEA culture.

For molecular identification of *Aspergillus* sp. SVUAw9, DNA was extracted following CTAB method described by Moubasher et al. [54]. PCR was conducted at SolGent Co. (Yuseong-Gu, 34014, Daejeon, South Korea), using SolGent EF [55], and the Calmodulin gene (*CaM*) was amplified using the primer pair CL1 (GAA TTC AAG GAG GCC TTC TC) and CL2A (TTT TTG CAT CAT GAG TTG GAC) [56]. Using DNASTAR (version 5.05), a contiguous sequence of *Aspergillus* sp. SVUAw9 was analyzed. The most similar sequences to that of *Aspergillus* sp. SVUAw9’*CaM* sequence, were downloaded from GenBank. All sequences in this analysis were aligned by MAFFT (version 6.861b) [57]. The alignment gaps and weak uninformative characters were optimized by Block Mapping and Gathering with Entropy (BMGE) [58]. MEGA X (version 10.2.6) was used to conduct the maximum-likelihood (ML) and maximum-parsimony (MP) phylogenetic analyses [59], and the robustness of the most parsimonious trees was evaluated by 1000 replications [60]. Utilizing Modeltest 3.7’s Akaike information criterion (AIC), the optimum nucleotide substitution model for ML analysis was identified [61].

### Fermentation medium

Sucrose-free Cz broth supplemented with 1.0% oat spelt xylan was used as fermentation medium. The medium contained (g/L): NaNO_3_, 2.0; K_2_HPO_4_, 1.0; KCl. 0.5; MgSO_4_, 0.5; ZnSO_4_, 0.1; FeSO_4_, 0.1; and CuSO_4_, 0.005.

### Optimization of xylanase production in submerged fermentation (SmF)

Sucrose-free Czapek’s broth, supplemented with 1.0% (w/v) oat spelts xylan as the exclusive carbon source, served as the fermentation medium [38, 39]. To enhance xylanase production under two-factor-at-a-time (TFAT) conditions by *A. welwitschiae* SVUAw9, various cultural parameters were optimized, including the initial medium pH (3.0, 4.0, 5.0, 6.0, 7.0, 8.0, 9.0, 10.0), incubation temperature (10, 15, and 20 °C), supplementary nitrogen sources (NaNO_3_, (NH_4_)_2_SO_4_, NH_4_Cl, peptone, yeast, and beef extract; each at 0.2%; (w/v), and the incubation duration (1–10 days) [38, 62]. Citrate buffer (pH 3.0–6.0), phosphate buffer (pH 7.0–8.0), and glycine/NaOH buffer (pH 9.0–10.0) were the buffers used to modify the pH. Two mL of spore suspension (containing 1.5 × 10^8^ spores/mL) derived from a seven-day-old culture of *A. welwitschiae* SVUAw9 were individually utilized to inoculate 50 mL of fermentation medium in 250 mL Erlenmeyer flasks. The flasks were subsequently incubated under shaking conditions at 150 rpm. Each experiment was conducted in triplicates.

### Xylanase production from agro-industrial residues

Five different agro-industrial residues were chosen for cold-active xylanase production in solid state fermentation (SSF): bean straw (BS), corn cobs (CC), date palm leaves (DPL), rice husk (RH), and sugarcane bagasse (SB). Public marketplaces in the Egyptian Governorates of Assiut and Qena supplied all the substrates. Subsequent to cleaning with distilled water, the materials were pulverized into particles capable of passing through a 2 mm sieve, and then subjected to oven drying at 50 °C until a stable weight was achieved [39].

### Xylanase production in solid state fermentation (SSF)

Five g of each agro-industrial residue (BS, CC, DPL, RH, and SB) were placed in triplicate in 250 mL Erlenmeyer flasks. Ten mL of the fermentation medium were employed to hydrate each sample, followed by the addition of distilled water to achieve an 80% moisture level. The xylanase optimal parameters obtained from SmF experiment were applied. pH was modified to 7.0 for the fermentation process, using ammonium chloride as nitrogen source. After autoclaving at 121 °C for 20 min, a 5 mL suspension containing 1.5 × 10^8^ spore/mL that was obtained from a 7-day-old culture of *A. welwitschiae* SVUAw9 was used to inoculate each flask. The flasks were then incubated at 10 °C for 15 days in a static environment [39]. After the incubation period, a double cheesecloth filter was employed to filter the fermented slurry after the contents of the flasks were separately collected in 100 mL of 50 mM phosphate buffer (pH 7.0). The protein-containing supernatants were then obtained by centrifugation (10,000 rpm at 4 °C for 10 min) and were then utilized as xylanase source. The experiment was conducted in triplicates.

### Xylanase assay and estimation of protein content

Xylanase activity was determined by combining 0.9 mL of 1.0% (w/v) oat spelts xylan, produced in 50 mM phosphate buffer (pH 7), with 0.1 mL of the protein-containing supernatant. The reaction mixture was thereafter incubated for 10 min at 10 °C. The reaction ended with the addition of 2.0 mL of dinitrosalicylic acid (DNS) reagent [63], thereafter boiled for 15 min. The absorbance of the sample (3.0 mL) was measured at 540 nm (UV-visible spectrophotometer; T80+; Leicestershire, UK), and the concentration of the reducing sugar was quantified using xylose standard curve, after which the xylanase activity was determined. One unit of xylanase activity is defined as the amount of enzyme that liberates 1.0 µmol of xylose per mL per minute under the standard assay conditions [64]. The protein concentration was assessed using the method outlined by Lowry et al. [65] using the standard curve of bovine serum albumin (BSA). The experiment was conducted in triplicates.

### Xylanase purification

#### Precipitation of total protein

Once the xylanase enzyme attained its optimum yield under its ideal conditions for production, the protein-containing supernatant was obtained by centrifugation (10,000 rpm for 15 min at 4 °C). While the cold absolute ethyl alcohol (−25 °C) was being slowly added, the protein-containing supernatant was being swirled gently at 4 °C [66] to precipitate the total protein. After precipitation, the protein was isolated by centrifugation, lyophilized, and then utilized in the subsequent purification steps.

#### Trilite MC 08 cation exchange column

Pre-activated Trilite MC 08 cation exchange resin was placed inside a glass column (60 cm × 3.6 cm), generating a bed volume of 400 cm^3^. Twenty mL of the crude xylanase sample were loaded to the ion exchange column. The bound proteins were eluted at 4 °C using 100 mM sodium citrate buffer (pH 4.0) and gradient NaCl concentrations ranging from 0 to 1.5 M, at a flow rate of 0.5 mL/min. Measurements of protein content and xylanase activity were made in fractions of 6.0 mL that were collected. In subsequent purification stages, the most active fractions were combined, concentrated, and used in further purification steps.

#### Sephacryl S 200 column

Sephacryl S 200 gel with a bed volume of 100 cm^3^ was placed inside a glass column (40 cm ×2.4 cm). Five mL of the xylanase fraction were loaded into the column. The bound proteins were eluted at 4 °C using 100 mM sodium citrate buffer (pH 4.0) at a flow rate of 0.5 mL/min. Measurements of protein content and xylanase activity were made in fractions of 6.0 mL that were collected. The most active fractions were combined and lyophilized.

#### Determination of xylanase molecular weight by SDS-PAGE

A 0.1 g of the lyophilized xylanase was dissolved in 100 µL of 20 mM Tris/HCl, pH 7.4 (Invitrogen, USA), which included 2.5% (w/v) bromophenol blue (tracking dye), 4.0% (w/v) SDS, 20% glycerol, and 10% (w/v) 2-mercaptoethanol. The protein samples were then heated at 100 °C for 5 min to ensure complete denaturation. After cooling briefly, the samples were loaded onto a 12% (w/v) SDS polyacrylamide gel. Electrophoresis was carried out at 100 mA and 150 V for 45 min at room temperature. After completing the electrophoretic run, protein bands were visualized by staining with Coomassie Brilliant Blue (R-250) and destained overnight with 7% (v/v) glacial acetic acid after documentation [67]. The gel was subsequently photographed using the Quantity One application (Version 4.6.2).

#### Effect of pH and temperature on the activity of pure xylanase

A 0.01 g of the purified xylanase was incubated at temperatures ranging from 5 to 35 °C with 0.01 g of oat spelts xylan (both dissolved in 1.0 mL of buffer solution; pH 3–11) to ascertain the optimal pH and temperature for the pure xylanase.

#### Effect of addition of some chemicals on xylanase activity

At the optimum pH and temperature, the effect of some chemicals on the activity of the purified xylanase was estimated [38]. NaCl, KCl, Ca (OH)_2_, Co (NO_3_)_2_, Ni SO_4_, CuSO_4_, FeSO_4_, MnSO_4_, MgSO_4_, ZnSO_4_, Phenylmethylsulfonyl fluoride (PMSF), ethylenediaminetetraacetic acid (EDTA), and sodium dodecyl sulphate (SDS) were investigated each at 5.0 mM. In the absence of the tested chemical, the enzyme activity was regarded as 100%.

#### Substrate specificity

The specificity of the purified xylanase was confirmed by evaluating its activity on xylans derived from oat spelts, corn cob, and maize stalks [68], in addition to beechwood xylan and Birchwood xylan (purchased from Sigma-Aldrich), and cellulosic substrates including carboxymethyl cellulose (CMC) and microcrystalline cellulose (Avicel). Substrates at 1.0% (w/v) (in a 0.05 M sodium citrate buffer at pH 4) were incubated with a proper diluted enzyme at 30 °C for 10 min. The reaction was then terminated by addition of 2.0 mL of DNS, and boiling for 10 min. Xylanase activity was calculated as described in the ‘Effect of pH and temperature on the activity of pure xylanase’ subsection (Materials and Methods).

#### Determination of kinetic constants (Km and Vmax)

To determine the kinetic constants Km and Vmax for the xylanase activity, an experiment was performed with oat spelts xylan at substrate concentrations ranging from 10 to 100 mg/mL. Microsoft Excel performed the necessary linear regression for Lineweaver-Burk plot [69].

### Statistical analysis

After conducting the initial investigation three times, the mean and standard deviation (SD) were used to express all data. The statistical significance analysis was conducted [70], and the significance level was set at *p* ≤ 0.05.

## Results

### Morphological and molecular identification of*Aspergillus* sp. SVUAw9

**Fig. 1:**
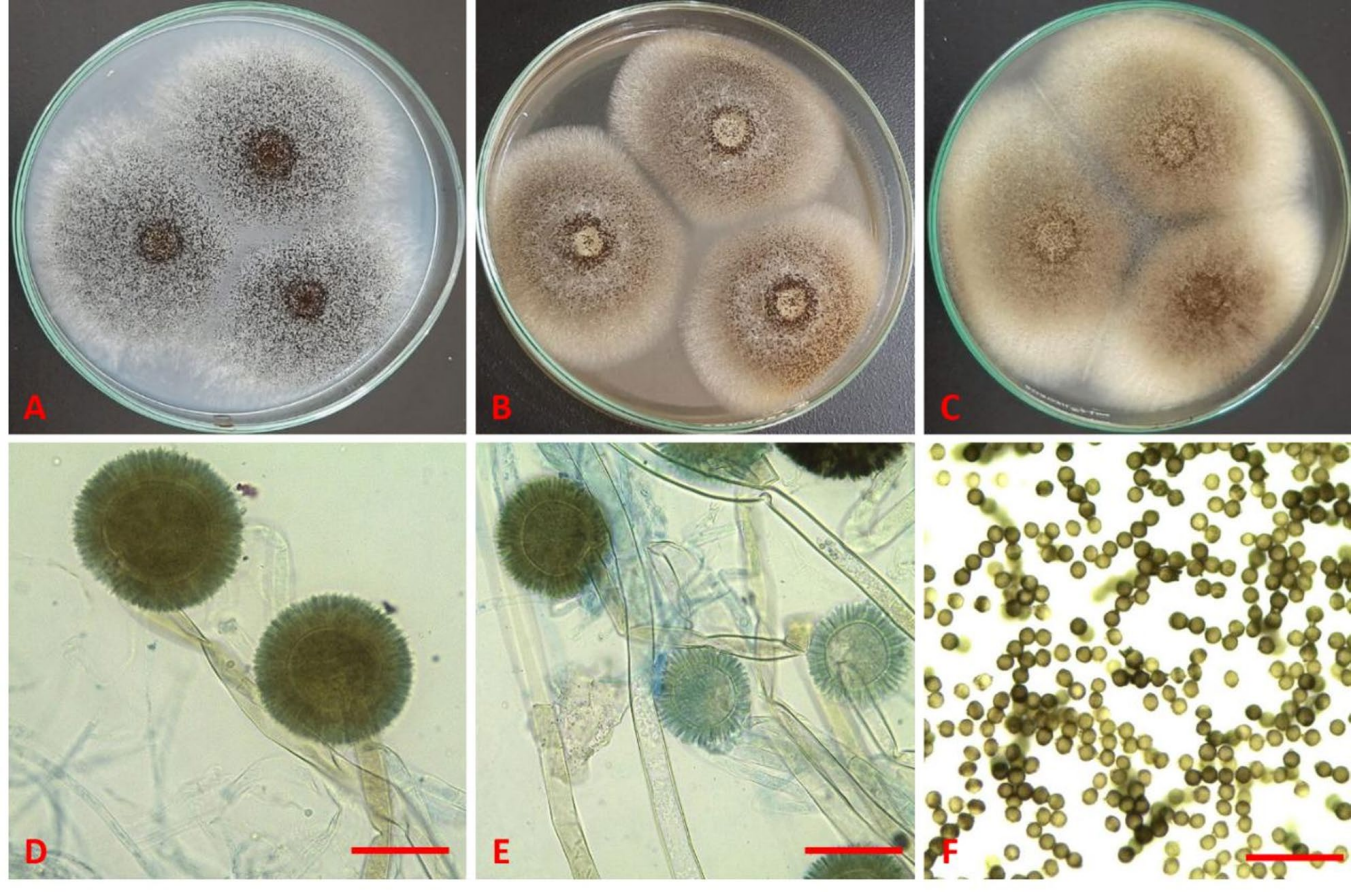
***Aspergillus welwitschiae*** SVUAw9: Seven-day-old colonies on **A** Cz **B** MEA and **C** CYA at 25 °C **D–E** conidiophores and conidial heads **F** conidia (scale bars: D–E = 50 μm; F = 20 μm)

The morphological similarities between the strain in this study and the type species allowed for its identification as *A. welwitschiae*. The conidiophores are long (up to 3000 μm × 15.0 μm), smooth-walled, hyaline. The fungus produces brown, finely to roughed, globose to subglobose conidia (3.0–4.2 μm), and globose to subglobose vesicles (up to 80 μm in size) that contain metulae (11–15 × 5–6 μm) and hyaline ampulliform phialides (5–11 × 3.0–3.5 μm) (Fig. 1).

**Fig. 2:**
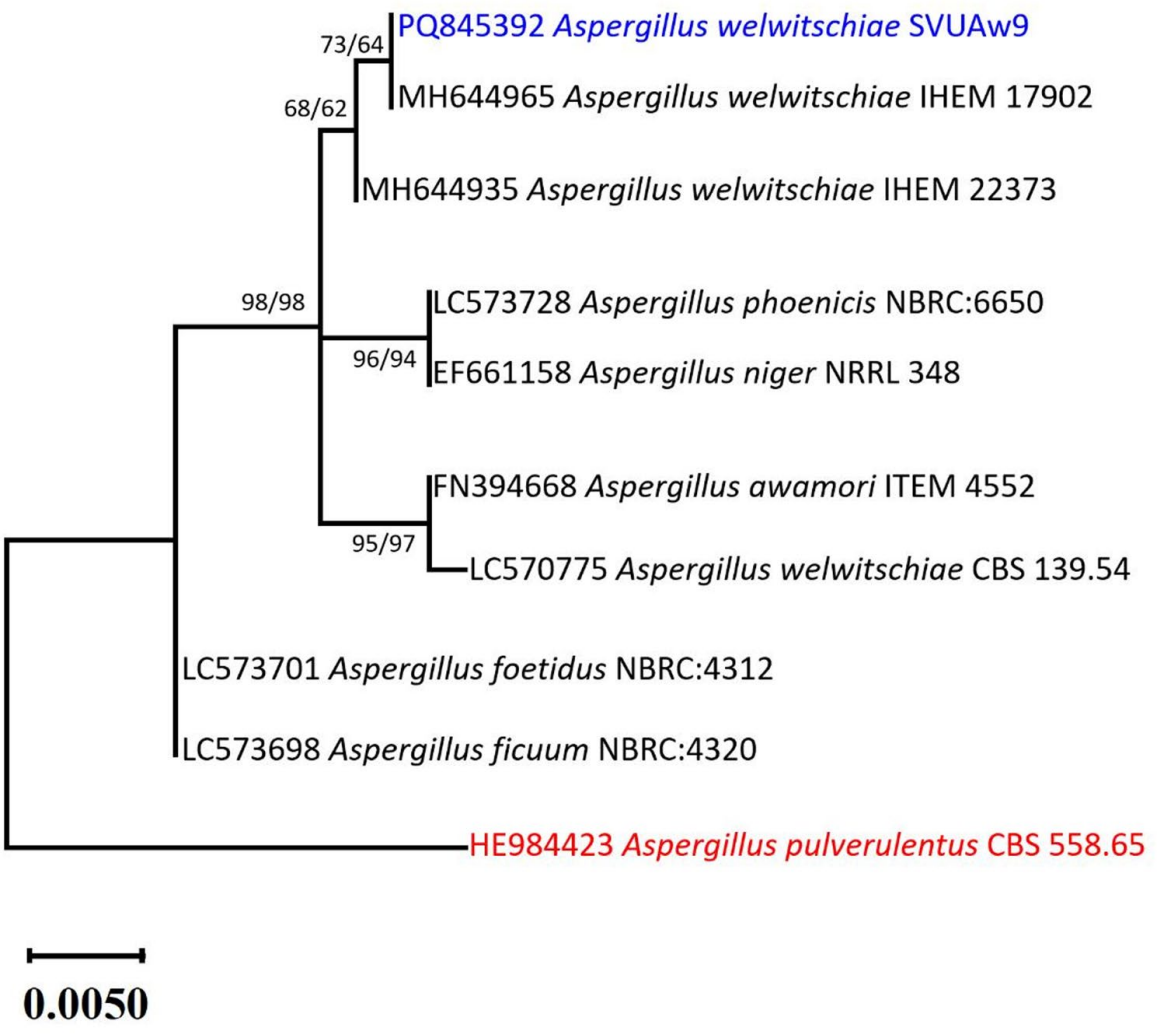
The maximum likelihood phylogenetic tree derived from a heuristic search (1000 replications) utilizing ML/MP analysis of *CaM* sequence of *A. welwitschiae* SVUAw9 (in blue) in relation to the most comparable species related to *Aspergillus*: section Nigri in GenBank. Bootstraps indicating support values for ML/MP ≥ 50% are displayed adjacent to the respective nodes. The tree is rooted to *Aspergillus pulverulentus* CBS 558.65 (in red)

To construct the phylogenetic tree, the *CaM* gene sequence of the isolate *A. welwitschiae* SVUAw9 was compared against sequences deposited in GenBank, and the most similar *CaM* sequences were retrieved for inclusion in the analysis. This dataset also incorporated the epitype strain *A. welwitschiae* CBS 139.54, which serves as the reference culture for the species. The placement of CBS 139.54 on a separate branch in the *CaM*-based tree is consistent with recent phylogenomic studies demonstrating substantial intra-specific divergence within *A. welwitschiae*, including lineages showing a closer evolutionary relationship to *A. niger* and reflecting complex molecular identity within section Nigri. Additionally, previous work has shown that black aspergilli represent one of the most taxonomically challenging fungal groups, and single-gene markers such as *CaM* may not fully resolve cryptic population structure. Therefore, the observed branching of CBS 139.54 reflects known genomic heterogeneity within the species rather than an artifact of the analytical method. Phylogenetically, the strain clustered with other *A. welwitschiae* strains in the corresponding clade, with bootstrap support of 68% under ML and 62% under MP, indicating moderate confidence in this placement (Fig. 2).

### Optimization of xylanase production

**Fig. 3:**
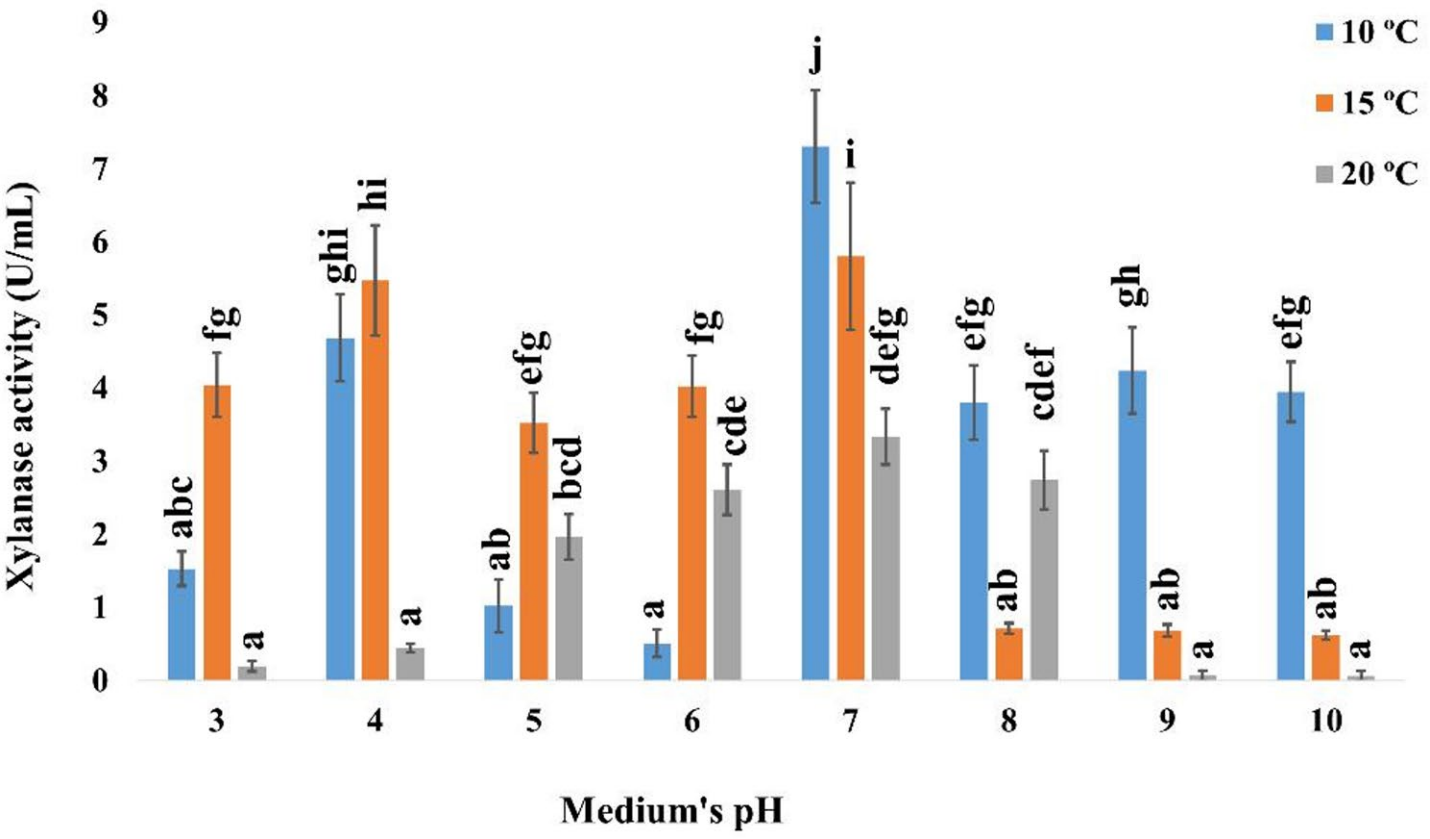
Effect of pH and temperature on the production of cold-active xylanase by *A. welwitschiae* SVUAw9 The data are displayed as Mean ± SD (*n* = 3). Different superscript letters indicate statistically significant differences at *p* ≤ 0.05 (one-way ANOVA with Duncan’s test). Means sharing a common letter (including overlapping letters such as ab) are not significantly different from one another

**Fig. 4:**
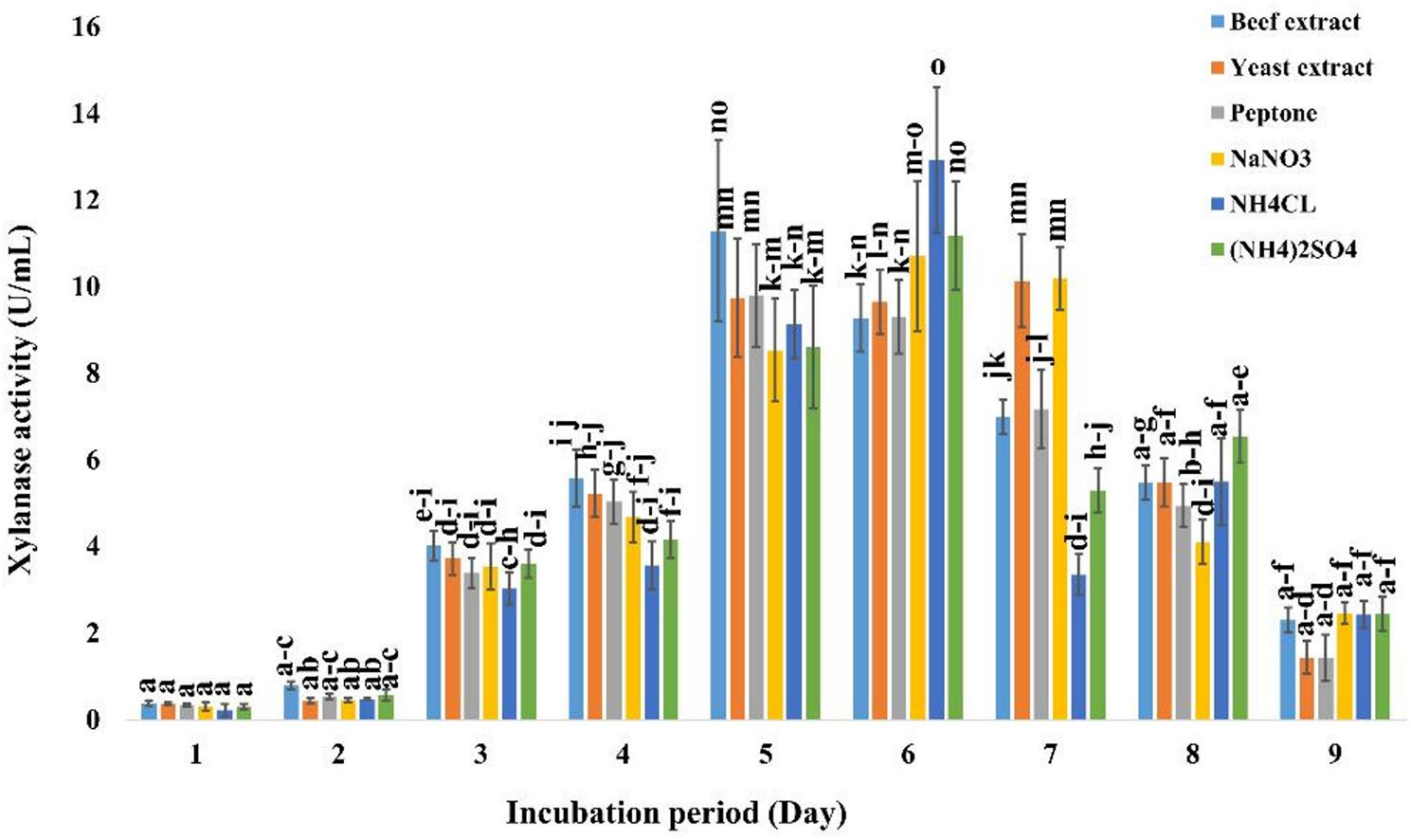
Effect of nitrogen source on the production of cold-active xylanase by *A. welwitschiae* SVUAw9 The data are displayed as mean ± SD (*n* = 3). Different superscript letters indicate statistically significant differences at *p* ≤ 0.05 (one-way ANOVA with Duncan’s test). Means sharing a common letter (including overlapping letters such as ab) are not significantly different from one another

The influence of pH range of 3.0–10.0 on xylanase production by *A. welwitschiae* SVUAw9 was evaluated within a pH range of 4.0 to 10.0 at temperatures between 10 and 20 °C. At pH 7.0 and 10 °C, xylanase had its peak activity of 7.3 ± 0.77 U/mL (Fig. 3). The activity rose to 12.92 ± 1.68 U/mL following six days of incubation with ammonium chloride as the nitrogen source (Fig. 4).

### Xylanase production in SSF

**Fig. 5:**
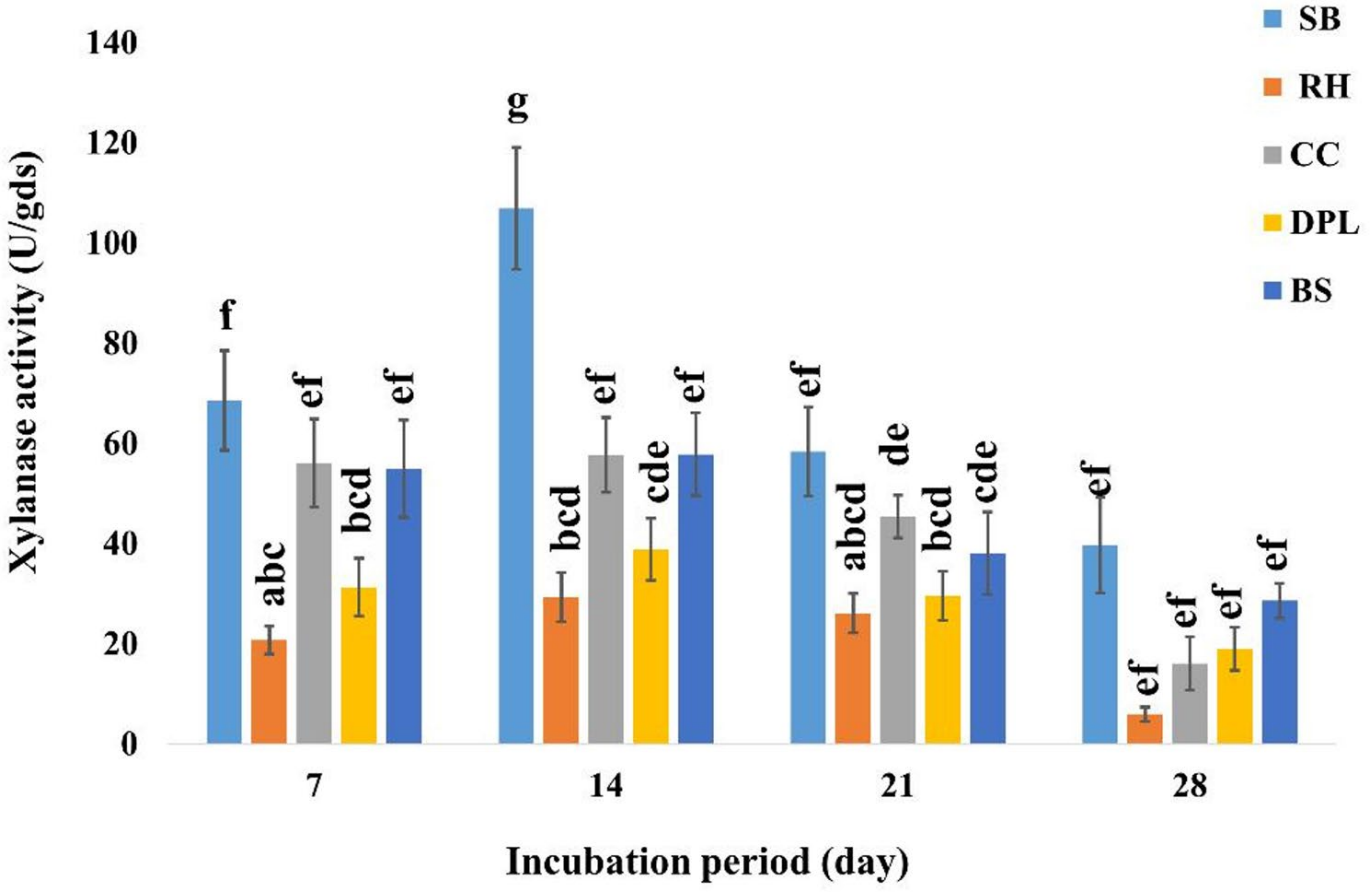
Xylanase production from some agro-industrial residues (bean straw (BS), corn cobs (CC), date palm leaves (DPL), rice husk (RH), and sugarcane bagasse (SB). by *A. welwitschiae* SVUAw9 at different days. The data are displayed as Mean ± SD (*n* = 3). Different superscript letters indicate statistically significant differences at *p* ≤ 0.05 (one-way ANOVA with Duncan’s test). Means sharing a common letter (including overlapping letters such as ab) are not significantly different from one another

Various lignocellulosic wastes were utilized as substrates for the economical and environmentally sustainable production of xylanase. *A. welwitschiae* SVUAw9 successfully produced xylanase through solid-state fermentation and fermented all utilized waste materials at varying incubation durations, albeit in differing amounts. The ideal period was 14 days, with sugarcane bagasse as the most effective substrate, generating 106.93 ± 12.1 U/gram dry substrate (gds), followed by bean straw, corn cob, and date palm leaves which produced 57.82 ± 8.27, 57.75 ± 7.44, 38.87 ± 6.15 U/gds xylanase, respectively. Conversely, the substrate exhibiting the lowest production was rice husk, producing 29.32 ± 4.88 U/gds (Fig. 5).

### Xylanase purification

**Fig. 6:**
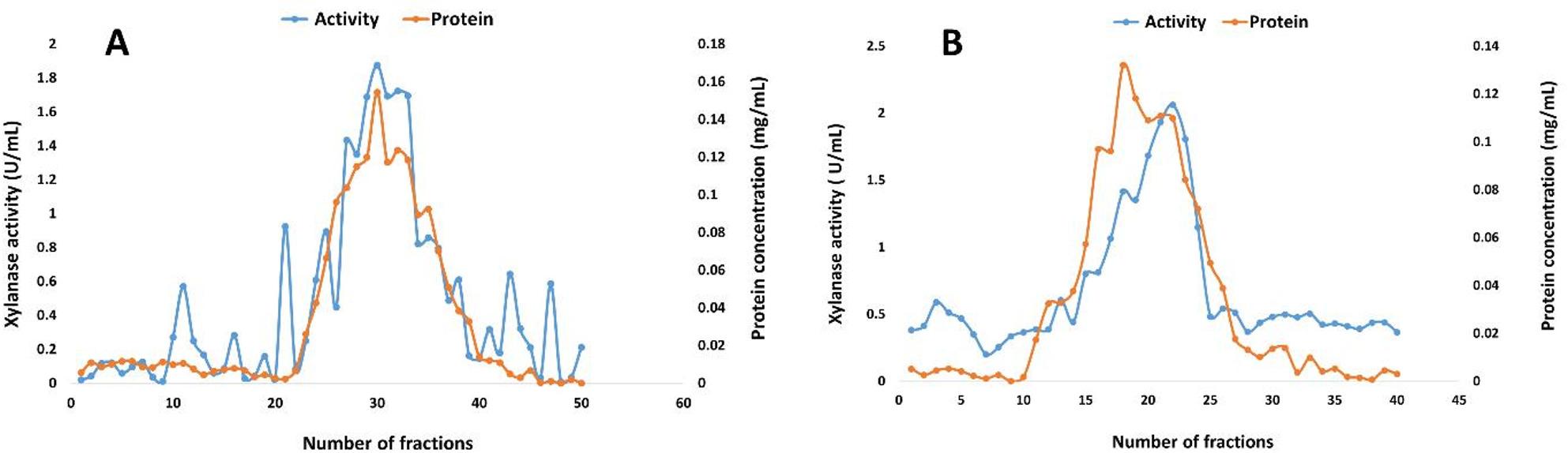
Elution diagram of xylanase produced by *A. welwitschiae* SVUAw9 using **A** Trilite MC 08 cation exchange resin **B** Sephacryl S 200 size exclusion gel

The protein-containing supernatant from a 14-day solid-state fermentation underwent a four-step purification process comprising centrifugation at 10,000 rpm, ethanol precipitation at −25 °C, cation exchange using Trilite MC 08 (Fig. 6A), and size exclusion chromatography with Sephacryl S 200 (Fig. 6B). Following the processing of the two columns, the specific activity of the purified xylanase was enhanced by 75.87-fold, achieving 145 U/mg with a yield of 5.45%. Table 1 presents the consolidated statistics regarding the purification of xylanase.

### Molecular weight determination by SDS-PAGE

**Fig. 7:**
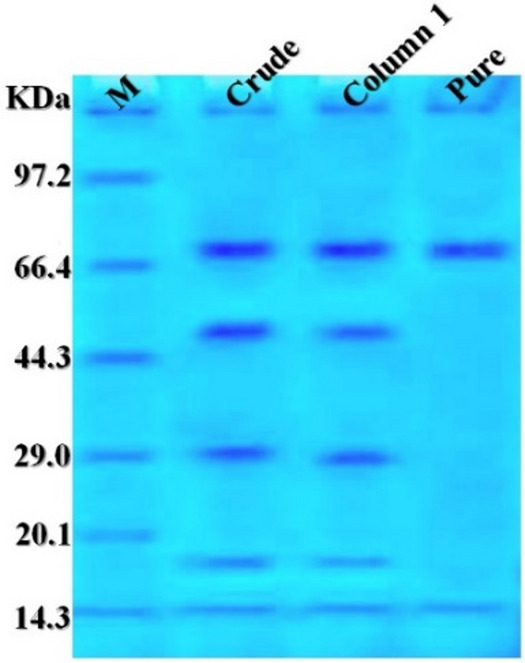
SDS-PAGE of the purified xylanase produced by *A. welwitschiae* SVUAw9. Column 1: Standard marker (M); Column 2: Crude xylanase; Column 3: Xylanase from Trilite MC 08 column; and Column 4: Xylanase from Sephacryl S 200 column

SDS-PAGE results revealed that the molecular weight of the purified xylanase was approximately 71 kDa (Fig. 7).

**Table 1:** Purification profile of xylanase produced from sugarcane bagasse by *A. welwitschiae* SVUAw9 in SSF The data are displayed as mean ± SD (*n* = 3). Different superscript letters indicate statistically significant differences at *p* ≤ 0.05 (one-way ANOVA with Duncan’s test). Means sharing a common letter (including overlapping letters such as ab) are not significantly different from one another

Purification steps	Volume (mL)	Activity (U/mL)	Total activity (U)	Protein content (mg/mL)	Total protein (mg)	Specific activity (U/mg)	Yield (%)	Fold
Fermentation medium	2000	0.904 ± 0.05^a^	1808 ± 200^c^	0.473 ± 0.02^d^	946 ± 40^b^	1.911	100	1
Cold absolute ethanol	50	10.62 ± 0.84^d^	531 ± 42^b^	0.235 ± 0.01^c^	11.75 ± 0.5^ab^	45.2	29.37	23.65
Trilite MC 08	18	7.1 ± 0.5^c^	127.8 ± 9^a^	0.064 ± 0.004^b^	1.152 ± 0.072^a^	110.93	7.0	58.0
Sephacryl S 200	20	4.93 ± 0.32^b^	98.6 ± 6.4^a^	0.034 ± 0.001^a^	0.68 ± 0.02^a^	145	5.45	75.87

### Optimum pH and temperature for the pure xylanase

**Fig. 8:**
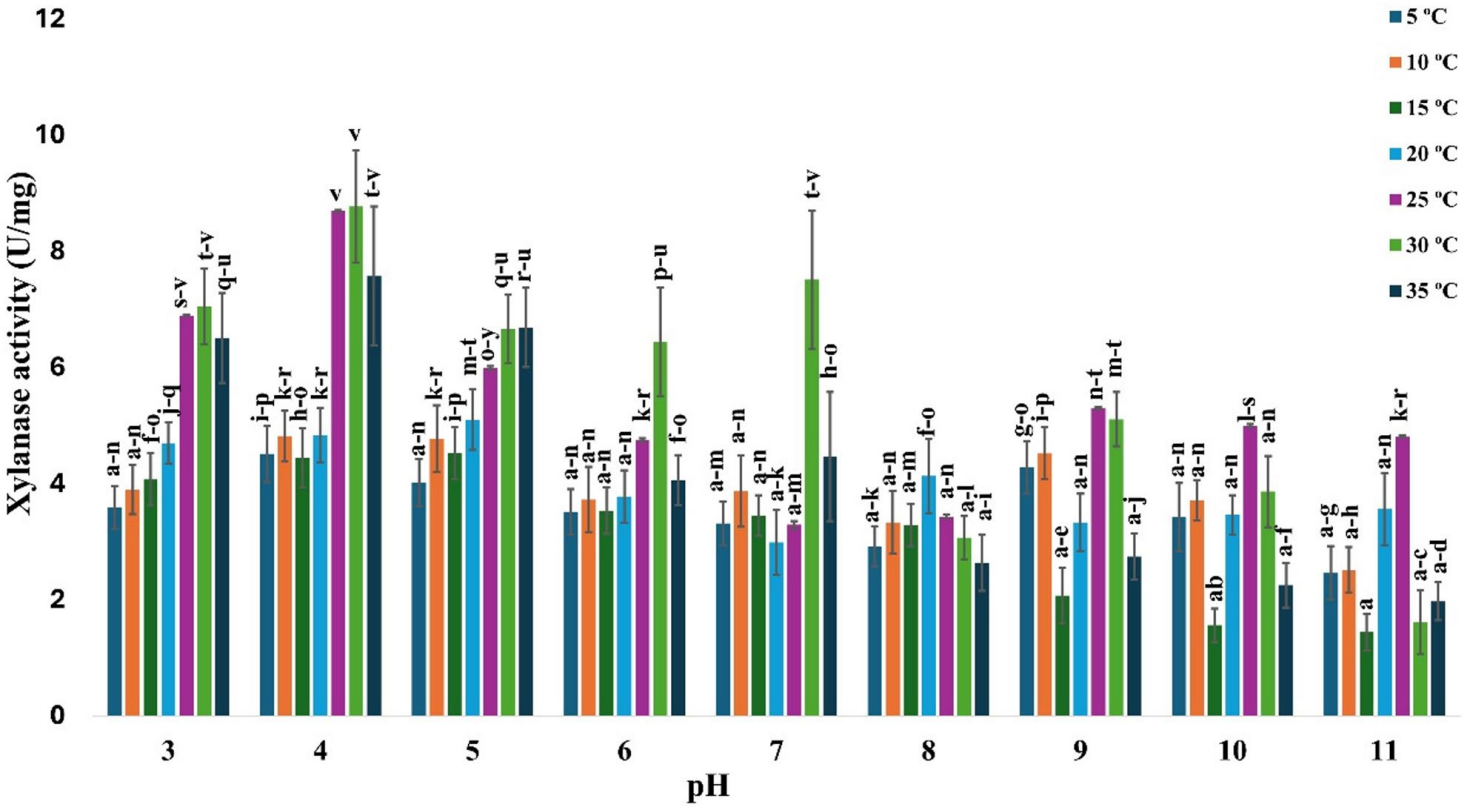
Determination of the optimal pH and temperature for the purified xylanase produced by *A. welwitschiae* SVUAw9. The data are displayed as mean ± SD (*n* = 3). Different superscript letters indicate statistically significant differences at *p* ≤ 0.05 (one-way ANOVA with Duncan’s test). Means sharing a common letter (including overlapping letters such as ab) are not significantly different from one another

The optimal pH and temperature for enzyme activity were evaluated using experiments conducted at different pH levels (3–11) and temperatures (5–35 °C). The maximum specific activity of the purified xylanase was achieved at pH 4.0 and 30 °C, resulting in 156.46 ± 12 U/mg (Fig. 8).

### Effect of addition of some chemicals on xylanase activity

The xylanase activity was significantly enhanced by the addition of 5 mM Co (NO_3_)_2_, MnSO_4_, and NiSO_4_, resulting in increases of 152.94 ± 11.54, 152.94 ± 9.58, and 134.12 ± 7.66% in residual activity, with specific activities of 239.3 ± 18, 239.3 ± 15, and 209.84 ± 12 U/mg, respectively. The addition of 5 mM of Ca (OH)_2_, MgSO_4_, and FeSO_4_ resulted in a moderate influence on xylanase activity, yielding specific activities of 182.23 ± 16, 182.23 ± 14, and 184.0 ± 12 U/mg, along with residual activities of 116.47 ± 10.22, 116.47 ± 8.94, and 117.65 ± 7.66%, respectively (Table 2).

**Table 2:** Effect of addition of metal ions and selected chemicals on the activity of the purified xylanase produced by *A. welwitschiae* SVUAw9. The data are displayed as mean ± SD (*n* = 3). Different superscript letters indicate statistically significant differences at *p* ≤ 0.05 (one-way ANOVA with Duncan’s test). Means sharing a common letter (including overlapping letters such as ab) are not significantly different from one another

Chemical added (5 mM)	Specific activity (U/mg)	Residual activity (%)
Control	156.46 ± 12^de^	100 ± 7.66^de^
NaCl	62.6 ± 4^bc^	70.59 ± 2.55^bc^
KCl	128.85 ± 9^cd^	82.35 ± 5.75^cd^
Ca (OH)_2_	182.23 ± 16^ef^	116.47 ± 10.22^ef^
MgSO_4_	182.23 ± 14^ef^	116.47 ± 8.94^ef^
FeSO_4_	184.0 ± 12^ef^	117.65 ± 7.66^ef^
Co (NO_3_)_2_	239.3 ± 18^g^	152.94 ± 11.54^g^
ZnSO_4_	62.6 ± 4^a^	40 ± 2.55^a^
MnSO_4_	239.3 ± 15^g^	152.94 ± 9.58^g^
CuSO_4_	147.26 ± 8^d^	94.12 ± 5.11^d^
NiSO_4_	209.84 ± 12^fg^	134.12 ± 7.66^fg^
EDTA	101.24 ± 6^bc^	64.71 ± 3.83^bc^
SDS	147.26 ± 8^d^	94.12 ± 5.11^d^
PMSF	92.0 ± 6^ab^	58.82 ± 3.83^ab^

### Substrate specificity

**Fig. 9:**
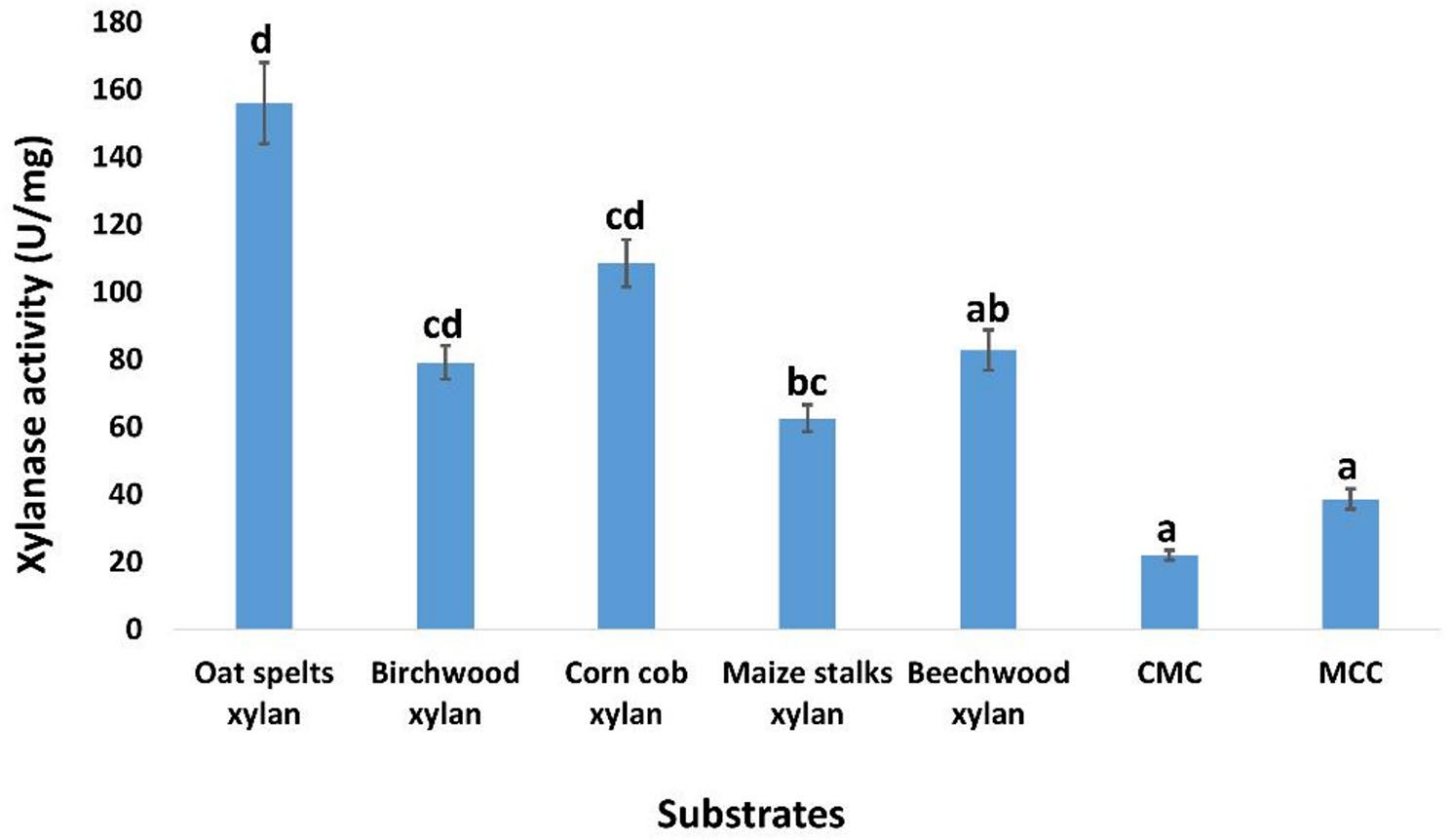
Substrate specificity of the pure xylanase using various types of xylans. The data are displayed as Mean ± SD (*n* = 3). Different superscript letters indicate statistically significant differences at *p* ≤ 0.05 (one-way ANOVA with Duncan’s test). Means sharing a common letter (including overlapping letters such as ab) are not significantly different from one another

The specificity of the pure xylanase was evaluated at pH 4.0 and 30 °C using various types of xylan, CMC and MCC. The purified xylanase demonstrated peak activity of 156.46 ± 12 U/mg with xylan sourced from oat spelts. Xylan derived from corn cobs, beechwood, Birchwood, and maize stalks exhibited specific activity of 108.6 ± 7, 82.8 ± 6, 79.15 ± 5, and 62.6 ± 4 U/mg, respectively. The minimal xylanase activity demonstrated with CMC and MCC as substrates, yielding 22 ± 1.5 and 38.65 ± 3 U/mg of specific activity, respectively (Fig. 9).

### Determination of*Km* and*Vmax*

**Fig. 10:**
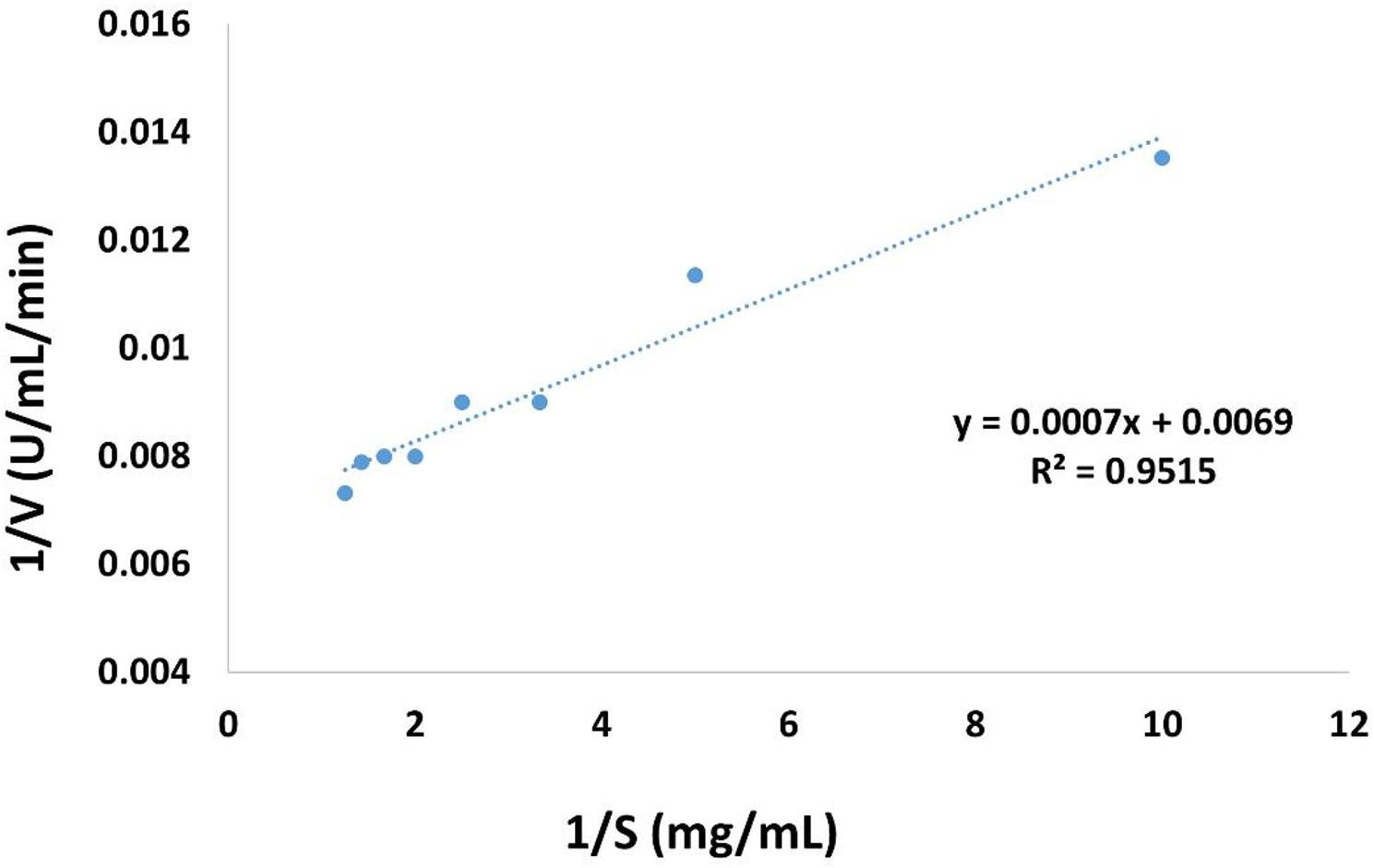
Lineweaver–Burk plot for the purified xylanase produced by *A. welwitschiae* SVUAw9

The kinetic parameters of the purified xylanase, *Km* and *Vmax*, were determined from the Lineweaver–Burk plot and were found to be 0.1 ± 0.005 mg/mL and 144.9 ± 7.14 µmol/min, respectively (Fig. 10).

## Discussion

The purpose of this work was to identify cold-active xylanase-producing fungi, optimize fermentation conditions, extract large quantities of xylanase from sugarcane bagasse, and purify it. *A. welwitschiae* SVUAw9, identified as the most efficient xylanase‑producing isolate, was initially evaluated under submerged fermentation to optimize the cultural parameters before being applied in solid‑state fermentation for high‑yield enzyme production. Xylanolytic enzymes have gained significant attention due to their potential applications in biorefinery processes and several industries, including textiles, paper and pulp, animal feed, bread, and fruit juice [71, 72]. In nature, xylanases are widely present in both eukaryotic and prokaryotic organisms. They are also present in bacteria and fungi from distinct environments [73, 74]. A multitude of mesophilic, thermophilic, and hyperthermophilic xylanases have been documented; Despite their biotechnological potential, psychrophilic and psychrotrophic xylanases have remained comparatively underexplored [13, 42, 75, 76]. To date, only a few cold-active xylanases from psychrophilic bacteria and fungi have been discovered, isolated, and subjected to detailed characterization, as research on xylanolytic systems adapted to low-temperature environments remains comparatively limited [77–79]. Although numerous mesophilic and thermophilic microorganisms have been extensively investigated for their xylanase-producing capabilities, microorganisms inhabiting permanently cold ecosystems—such as polar soils, Antarctic marine habitats, and other psychrophilic niches—are still relatively underexplored [43, 80, 81]. Consequently, the number of well-characterized cold-active xylanases remains small, and each newly identified psychrophilic strain represents a valuable addition to the current understanding of xylan degradation under low-temperature conditions. The scarcity of such studies underscores the importance of continued exploration of cold environments to uncover novel cold-adapted xylanases with unique structural and catalytic properties, particularly those offering high activity and stability at low temperatures, which are advantageous for various industrial processes.

By modifying the growth conditions for xylanase production by applying the TFAT methodology, this study emphasizes the psychrophilic features of *A. welwitschiae* SVUAw9. In this study, after six days of incubation at pH 7.0 and 10 °C with ammonium chloride as the nitrogen source, xylanase had its peak activity of 12.92 ± 1.68 U/mL in SmF. A medium’s nutritional components and cultural conditions have a major impact on xylanase production. Several studies included pH, temperature, incubation period, and nitrogen sources as physical and chemical characteristics that affect xylanase production. Ameen [38] demonstrated that *Aspergillus fumigatus* produced xylanase optimally under mesophilic conditions, with maximum activity occurring at higher temperature (50 °C). In contrast, *A. welwitschiae* SVUAw9 produced a cold-active xylanase with optimal activity at 30 °C, production at 10 °C, underscoring its distinct psychrotrophic adaptation. Brandelli et al. [82] emphasized that most fungal hydrolases, including keratinases and xylanases, typically exhibit mesophilic or thermophilic activity profiles, with temperature optima well above ambient levels. Our findings diverge from this general trend, positioning *A. welwitschiae* SVUAw9 among the relatively rare cold-active fungal enzyme producers, particularly valuable for low-temperature industrial processes.

Similarly, Bocchini et al. [83] optimized xylanase production by *Bacillus circulans* D1 and reported temperature optima around 50 °C and enhanced production under alkaline pH, distinctly different from the neutral pH (7.0) and 10 °C required for optimal production by *A. welwitschiae* SVUAw9. This contrast highlights fundamental differences in thermal tolerance and metabolic regulation between bacterial thermotolerant systems and cold-adapted fungal isolate. Cunha et al. [84] optimized xylanase production by *Aspergillus foetidus* using soybean residues and found that production was maximized under mesophilic temperatures, consistent with the behavior of most *Aspergillus* species. In contrast, the ability of *A. welwitschiae* SVUAw9 to produce high xylanase levels at low temperature and utilize sugarcane bagasse efficiently under solid-state fermentation demonstrates an uncommon metabolic flexibility and superior performance in energy-efficient fermentation systems. Taken together, the comparisons with these studies highlight the distinctiveness of *A. welwitschiae* SVUAw9, particularly its cold-activity, higher enzyme molecular weight, and ability to convert agro-residues under low-temperature conditions. These characteristics position this strain as a valuable candidate for industrial applications requiring reduced energy input, including biobleaching, cold-wash detergents, food processing, and cold-climate biomass hydrolysis.

The extensive production of xylanase is economically impractical when utilizing pure xylan because of its high cost. As a result, many cost-effective agricultural byproducts were examined for xylanase production. This work demonstrated that the utilization of sugarcane bagasse in solid-state fermentation (SSF) allowed *A. welwitschiae* SVUAw9 to attain peak xylanase activity of 106.93 ± 12.1 U/gds under optimal conditions of pH 7.0, following 14 days of incubation at 10 °C, with ammonium chloride serving as the nitrogen source. Filamentous fungi, especially *Aspergillus* species, are particularly noteworthy due to their superior productivity compared to other filamentous fungi and bacteria [38, 85]. Comparing data of enzyme activity across multiple studies may prove difficult due to minor methodological discrepancies. Consequently, one must take prudence while recognizing similarities. Regarding this, *Penicillium oxalicum* produced 38.9 U/gds of xylanase from wheat bran [86]. Multiple strains of *A. flavus* engaged in SSF, producing xylanase at diverse concentrations (191–738 U/gds) from wheat bran [87]. The production of xylanase through SSF has been recorded by *A. niger* DFR-5. The peak activity of 2596 IU/gds was attained in a medium of wheat bran and soybean cake in a 70:30 ratio [88]. *Aspergillus oryzae* utilized wheat bran in SSF to generate significantly elevated amounts of xylanase (2830.7 U/gds) [89]. *Aspergillus flavus* fermented wheat bran, sugarcane bagasse, and corn cob to produce the highest xylanase activity of 129.8 and 94.0 U/gds in SmF [90]. *A. tubingensis* FDHN1 exploited sorghum straw and had the highest xylanase activity of 615.5 ± 19.21 U/gds [91]. *A. flavus* produced a total of 32.4 U/gds. *Aspergillus niger*, *A. oryzae*, and *A. awamori* employed palm kernel cake and palm pressed fiber to produce xylanase activities of 18.8, 27.2, and 134.2 U/g, respectively [92]. *A. fumigatus* ITBCCL170 successfully fermented empty fruit bunches, yielding 236.30 U/g of xylanase [93]. The endophytic fungus *Colletotrichum boninense* yielded 383.63 U/mL of xylanase activity utilizing a sugarcane straw-bagasse mixture after 6 days at 28 °C [94]. *A. fumigatus* KSA-2 utilized wheat bran in SSF to produce 66.0 *±* 8.65 U/gds [38].

This study focused on the purification of the cold-active xylanase generated by *A. welwitschiae* SVUAw9, applying ethanol precipitation, cation exchange chromatography on a Trilite MC 08, and size exclusion gel chromatography using Sephacryl S-200. A yield of 5.45% and a specific activity of 145 U/mg resulted in a 75.87-fold purification. The present findings markedly exceed the xylanase produced by *A. tubingensis* TSIP9, which attained a specific activity of 8.48 U/mg and a purification factor of 7.4 with a yield of 9.07% via a two-step column chromatography employing DEAE-Sepharose anion exchange resin and gel filtration on Sephadex G-75 [95]. However, the xylanase produced by *Paecilomyces variotii* in the stirred tank bioreactor displayed a xylanase recovery of 34.47% and increased purity by a factor of 3.29. The purified enzyme fraction demonstrates a specific enzyme activity of 3968 U/mg protein utilizing wheat bran as substrate [96].

The maximum specific activity of the purified xylanase in this study was achieved at pH 4.0 at 30 °C, yielding 156.46 ± 12.3 U/mg of specific activity while retaining 50.98% (79.765 ± 10.8 U/mg) of its activity at 5 °C. Cold-adapted xylanase often exhibits a reduced optimal temperature and heightened catalytic activity at lower temperatures compared to its mesophilic counterparts. In accordance with other investigated cold-adapted enzymes, a new xylanase from the marine microbe *Zunongwangia profunda* exhibited maximum activity at pH 6.5 and 30 °C, retaining 23% and 38% of ideal activity at 0 °C and 5 °C, respectively [79]. The xylanase synthesized by *Sorangium cellulosum* demonstrated an optimal temperature range of 30–35 °C, with 33.3% activity at 5 °C and 13.7% activity at 0 °C [44].

Pure fungal xylanases typically possess molecular weights between 20 and 45 kDa [95, 97, 98], but bacterial xylanases demonstrate lower molecular weights of 23 to 26 kDa [99]. Acid xylanases exhibit a higher molecular weight and are classified within the GH10 family, while numerous basic xylanases, with a molecular weight of roughly 30 kDa, are categorized in the sequence-based glycoside hydrolase (GH) 11 family [100, 101]. Regarding this sense, the SDS-PAGE analysis revealed that the isolated xylanase in this work possessed a molecular weight of approximately 71 kDa. The xylanase derived from *A. tubingensis* TSIP9, possessing a molecular weight of around 40–45 kDa, displayed two bands on SDS-PAGE that aligned with those of commercial xylanase [95].

This study incorporated several metal ion solutions at a concentration of 5.0 mM into pure xylanase reaction, which demonstrated varying impacts on xylanase activity. Xylanase activity increased by 152.94 ± 9.58 and 152.94 ± 11.54% with Mn^2+^ and Co^2+^, respectively, 134.12 ± 7.66 with Ni^2+^, 116.47 ± 8.94 and 116.47 ± 10.22 with Mg^2+^ and Ca^2+^, respectively, and 117.65 ± 7.66 with Fe^2+^, whereas Na^+^, K^+^, Zn^2+^, Cu^2+^, EDTA, SDS, and PMSF induced inhibition to xylanase activity demonstrating residual activities of 70.59 ± 2.55, 82.35 ± 5.75, 40 ± 2.55, 94.12 ± 5.11, 64.71 ± 3.83, 94.12 ± 5.11, and 58.82 ± 3.83%, respectively compared to the control. In accordance with these results, Mg^2+^ increased the xylanase activity purified from *A. niger* US368 by 12%, presumably due to the structural stability provided by Mg^2+^ [102]. Ca^2+^ also enhanced xylanase activity from *A. niger* US368 [103]. The enzyme activity of the *Trichoderma harzianum* demonstrated a little enhancement of 1.8% with 2 mM Mn^2+^ [104]. The activity augmentation by Ca²⁺, Fe²⁺, and Mn²⁺ was similarly recorded for xylanase extracted from *Aspergillus tubingensis* TSIP9 [95]. Ameen [38] reported similar improvements in xylanase activity with the addition of some metal ions, noting a 28% rise in the specific activity of xylanase when 5 mM of Mn^+ 2^ was added to the xylanase’ reaction. These metals serve as cofactors in the enzyme-substrate reaction [105, 106], while calcium protects xylanase from proteolytic inactivation and heat denaturation [107].

Conversely, numerous prior studies have shown that some metal ions may significantly inhibit xylanase activity, especially in well-defined cold-active xylanases, due to irreversible interactions with the main polypeptide or side chains. In this regard, the activity of xylanase from *Sorangium cellulosum* So9733-1 was markedly diminished by Cu^2+^, Zn^2+^, Ni^2+^, Fe^2+^, Co^2+^, Ag^+^, Hg^2+^, EDTA, and SDS at concentrations of 1 or 10 mM [44]. Xylanase from *A. ficuum* AF-98 exhibited a 115.8% rise [108], but it was inhibited by Fe^2+^. The xylanase activity of *A. awamori* 2B.361 U2/1 was augmented by Mg^2+^ and inhibited by Cu^2+^ [109]. The xylanase activity of *Trichoderma inhamatum* was inhibited by Zn²⁺, Cu²⁺, PMS, SDS, and EDTA, while Mn²⁺, Mg²⁺, Co²⁺, and Ca²⁺ augmented xylanase activity [110]. The residual activity of Xyn27 was measured at 93.23% with EDTA and 83.07% with SDS [111].

In this study, the maximum substrate specificity of the pure xylanase for polysaccharide breakdown was assessed using possible substrates such as oat spelt xylan, corn cob xylan, birchwood xylan, maize stalks xylan, CMC, and MCC under the optimal circumstances (pH 4.0 and temperature of 30 °C). The peak activity (156.46 ± 12 U/mg) was determined with xylan from oat spelts, succeeded by maize cob xylan, whereas the minimal activity was recorded for CMC and MCC. Similar results were documented by de Sousa Gomes et al. [112] in their study on the purification of xylanases obtained from *Chrysoporthe cubensis*. The peak activity of purified xylanase was observed in *Bacillus cereus* [113], *Trichoderma inhamatum* [110], and *Caldicoprobacter algeriensis* [114] on oat spelt xylan, subsequently followed by beachwood xylan, with no activity detected against glycans such as carboxymethyl cellulose and cellobiose.

Xylanase specificity is fundamentally shaped by the chemical structure and complexity of the xylan substrates they act upon. Xylans vary widely across plant sources, differing in branching patterns, substituent groups, glycosidic linkages, degree of polymerization, and side-chain decorations, and these structural differences greatly influence how individual xylanases interact with them [115]. Thus, substrate complexity directly determines whether an enzyme performs sharp internal cleavage (endo-type) or shifts towards more terminal cleavage (exo-like behavior). Purified enzyme activity was found to be modest towards CMC and MCC, with the highest activity observed on oat spelt xylan, corncob, beechwood, birchwood, and maize stalk xylans, according to the current results. The pattern here is a perfect reflection of the material of the substrate. Consequently, the enzyme demonstrated almost no preference for substrates derived from cellulose and instead favored xylans with balanced substitution and improved solubility.

This study utilized different concentrations of oat spelt xylan at the optimal conditions to determine kinetic parameters of the pure xylanase. The Lineweaver–Burk plots revealed that the Km and Vmax for oat spelt xylan were 0.1 ± 0.005 mg/mL and 144.9 ± 7.14 µmol/min, respectively. Km is a constant that is invariant for each enzyme substrate interaction. A low Km signifies a strong substrate affinity, whereas a high Vmax suggests enhanced enzyme activity [116]. Tiwari et al. [117] reported that the Km and Vmax of *Bacillus paramycoides* T4 for birchwood xylan were 1.473 mg/mL and 654.017 µmol/min, respectively. Amel et al. [114] established that the kinetic parameters Km and Vmax for XYN35 were 1.33 mg/mL and 595 µmol/min, respectively, utilizing oat-spelt xylan as the substrate. Notably, the exceptionally low Km value (0.1 ± 0.005 mg/mL) for the purified xylanase in this study indicates a very high affinity of the enzyme for oat spelt xylan, which is superior to many previously reported fungal and bacterial xylanases.

## Conclusion

This work identified *A. welwitschiae* from sugarcane bagasse by sequencing the calmodulin (*CaM*) gene. It showed significant cold-active xylanase activity. The strain produced cold-active xylanase from agro-industrial residues under solid state fermentation. Sugarcane bagasse produced the most after 14 days at pH 7 and 10 °C with ammonium chloride as the nitrogen source, followed by bean straw, corn cob, and date palm leaves, while rice husk had the lowest output. Xylanase was purified using Trilite MC 08 and Sephacryl S-200 columns, and its molecular weight was determined as approximately 71 kDa. The maximum xylanase activity was achieved at pH 4.0 and 30 °C. Co (NO_3_)_2_, MnSO_4_, and NiSO_4_ increased enzyme activity. Km and Vmax for pure xylanase were determined as 0.1 ± 0.005 mg/mL and 144.9 ± 7.14 µmol/min, respectively. The pure xylanase degraded oat spelt, corn cob, beechwood, Birchwood, maize stalk, carboxymethyl cellulose (CMC), and microcrystalline cellulose (MCC).

### Novelty statement and technological relevance

This study provides a comprehensive characterization of a cold-active xylanase produced by *Aspergillus welwitschiae* SVUAw9, a strain isolated from sugarcane bagasse. The work stands out from previous publications in several key aspects. First, *A. welwitschiae* has rarely been reported as a producer of cold-active xylanase, and no earlier studies have described the combined optimization, purification, and kinetic evaluation of a cold-active xylanase from this species. Second, the enzyme is produced efficiently under solid-state fermentation using low-cost agro-industrial residues—particularly sugarcane bagasse—demonstrating a sustainable and economically favorable bioprocess. This is notable because most prior work on cold-active xylanases has centered on bacteria or marine fungi, while fungal cold-active xylanases remain comparatively underexplored. The purified enzyme exhibited high activity at low temperatures and acidic pH, a profile attractive for several industrial sectors. Cold-active xylanases can reduce energy inputs in bioprocesses, making them valuable in cold-wash detergents, juice clarification, saccharification of biomass in cold climates, biobleaching of pulp at reduced temperatures, and preservation-sensitive food processes. The enzyme’s broad substrate specificity—including activity toward multiple plant xylans—further highlights its potential for biomass valorization, especially in biorefineries seeking to convert agricultural waste into fermentable sugars or prebiotic xylooligosaccharides. Beyond its current applications, this study opens multiple avenues for future research. Structural and genomic analyses could reveal the molecular basis of its cold adaptation and unusually high molecular weight (71.13 kDa). Protein engineering—through directed evolution or domain truncation—may further enhance catalytic efficiency, thermostability, or metal ion tolerance. Additionally, scaling-up studies and integration into industrial bioprocesses would help establish its commercial potential. The discovery of *A. welwitschiae* SVUAw9 as an efficient producer of cold-active xylanase expands the repertoire of fungal cold-active enzymes and provides a promising platform for developing sustainable biotechnological applications.

## Data Availability

The dataset generated and/or analyzed during the current study is available in the GenBank: [*Aspergillus welwitschiae strain SVUAw9 calmodulin (CmdA) gene, partial - Nucleotide - NCBI*] (https:/www.ncbi.nlm.nih.gov/nucleotide/PQ845392.1?report=genbank&log$=nucltop&blast_rank=3&RID=JC6ZTN87014); accession number PQ845392.
